# First molecular characterization of *Echinococcus granulosus sensu stricto* genotype G1 from a common chimpanzee (*Pan troglodytes*) in the Kuwait Zoo

**DOI:** 10.1007/s11686-026-01422-x

**Published:** 2026-10-20

**Authors:** Osama M. E. El-Azazy, Amel G. Abouelella, Fatemah A.M. Aryan, John M. Kinsella, Laila M.A. Tahrani, Vasyl V. Tkach

**Affiliations:** 1 Veterinary Laboratories, PAAFR, P.O. Box 21422, Safat, Kuwait , 13075 Kuwait; 2 Department of Parasitology, Faculty of Veterinary Medicine, Zagazig University, Zagazig, Egypt; 3 Zoo, PAAFR, P.O. Box 21442, Kuwait, 13075 Kuwait; 4 Department of Science, College of Basic Education, PAAET, Kuwait, Kuwait; 5 Helm west Laboratory, 2108 Hilda Ave, Missoula, Montana 59801 USA; 6 Department of Biology, University of North Dakota, Starcher Hall, 10 Cornell Street Stop 9019, Grand Forks, ND 58202 USA

**Keywords:** *Echinococcus granulosus*, Non-human primate, Cystic echinococcosis, Common chimpanzee

## Abstract

**Purpose:**

Cystic echinococcosis (CE) is a cosmopolitan parasitic zoonosis with public health and economic significance. In this study, we report CE in uncommon host, a chimpanzee (*Pan troglodytes*) in the Kuwait Zoo with description of clinical and pathological findings.

**Methods:**

In September 2025, a 32-year-old adult female common chimpanzee (*Pan troglodytes*) died at the zoo of Kuwait after showing severe symptoms. Post-mortem examination revealed a handball-sized cyst embedded in the hepatic umbilical fissure, causing lesions. The content of the cyst was examined microscopically as well as by sequencing of the mitochondrial nad1 gene from one of the clonal protoscoleces found in the cyst.

**Results:**

The cyst proved to be *Echinococcus granulosus sensu stricto*, genotype G1. The clinical case of the infection is described in detail. DNA sequence was nearly identical to sequences of G1 genotype of *E. granulosus* available in GenBank.

**Conclusions:**

This report of CE in a chimpanzee in the zoo highlights the problem related to environmental contamination. The implication of dogs in environmental contamination and other aspects of CE epidemiology in Kuwait is discussed. This is the first report of genetically identified hydatid cyst of *E. granulosus sensu stricto* in Kuwait and the first genetically confirmed case of the genotype G1 in chimpanzee.

## Introduction

Echinococcosis is one of the most serious parasitic zoonoses with substantial public health and economic significance. It is caused by the larval stages of *Echinococcus* species, which differ in their morphology, biology and geographical distribution. *Echinococcus granulosus* is cosmopolitan and *E. multilocularis* is distributed in the northern hemisphere, while *E. vogeli* and *E. oligarthrus* are confined to the Neotropics [1, 2].

Based on genetic data, *E. granulosus sensu lato*, the causative agent of cystic echinococcosis (CE), comprises several genotypes that have traditionally been designated G1–G10 and are currently assigned to 5 distinct species within the *E. granulosus* species complex as follows: G1-G3 (*E. granulosus sensu stricto*), G4 (*E. equinus*), G5 (*E. ortleppi*), G6, G7 (*E*. *intermedius*), G8-G10 (*E. canadensis*). Among these species, *E. granulosus sensu stricto*, particularly the genotype G1 (sheep genotype), is the most common and responsible for most human CE cases worldwide [3, 4].

*Echinococcus granulosus* has a complex life cycle that includes canid definitive hosts while the intermediate hosts are mainly various herbivorous and omnivorous mammals, which harbor the larval stage (hydatid cyst). Although from the physiological viewpoint humans are suitable intermediate hosts, from the ecological viewpoint they are dead end hosts as they do not normally contribute to completion of the parasite’s life cycle. Likewise, non-human primates (NHPs) can also be involved in *Echinococcus* life cycle as intermediate hosts, but unlikely to serve as a source of infection for definitive hosts [2]. Cystic echinococcosis is endemic in Kuwait where the disease has been reported in humans and animals [5, 6]. However, to date no molecular analysis has been conducted to identify *Echinococcus* genotypes in the country. Herein, we describe a fatal case of CE in a common chimpanzee (*Pan troglodytes*) in the Kuwait Zoo with detailed clinical and pathological findings accompanied by molecular identification of the genotype.

## Case presentation

**Fig. 1:**
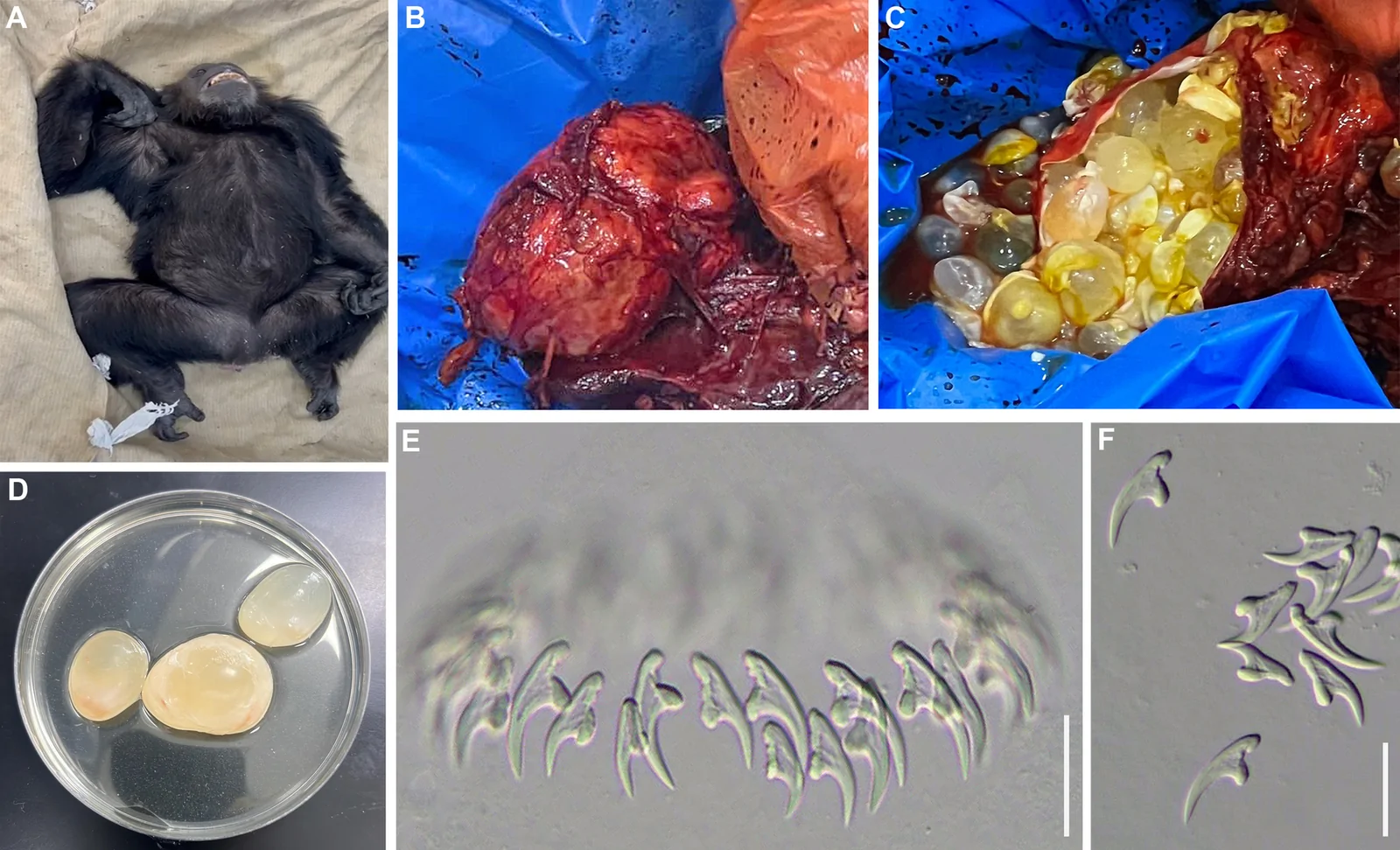
*Echinococcus granulosus* from common chimpanzee in the Kuwait Zoo. **A** Chimpanzee at advanced stages of disease; **B**, **C** Entire and ruptured cyst; **D** Separated internal cysts; **E**, **F** Hooks of protoscoleces

In September 2025, a 32-year-old adult female common chimpanzee died at the Kuwait Zoo. The animal shared the cage with an adult male chimpanzee. Its diet consisted of vegetables and fruits brought from the local market. The zoo records revealed that it was imported to Kuwait from Nigeria 25 years earlier. Since arrival, the chimpanzee has been kept in the zoo, and as far as it could be determined, remained healthy without showing a serious illness. However, according to the medical report, the animal showed three episodes of respiratory distress, the first one in November 2024 and the last one in July 2025. During the last six months before death, it became anorectic, depressed, lethargic with pale conjunctival mucous membranes, slightly distended abdomen and alopecia. At that time, our diagnosis was that the animal had gastrointestinal disturbances and bloat; liver tonic, stomachic and multivitamins were given without improvement. At the late stage of the disease course, the animal was in a miserable condition, showing difficulty breathing, while often gasping and teeth grinding in pain (Fig. 1A).

Post-mortem examination revealed the presence of a cyst approximately the size of a handball (Fig. 1B) embedded in the umbilical fissure of the liver, causing pressure on the bile duct, pyloric opening of the stomach and portal blood vessels. The liver appeared enlarged and congested, the bile duct and gall bladder were distended, and the subcutaneous fatty layer appeared deeply yellow in color, indicating severe jaundice.

When the large thick-walled cyst was incised, small thin-walled vesicles came out (Fig. 1C). Three of these fluid-filled vesicles (daughter cysts) (Fig. 1D) were sent to our laboratory for identification. Microscopic examination of the fluid and sediment from one daughter cyst revealed protoscoleces bearing a crown of characteristic hooks (Fig. 1E, F). One of the daughter cysts and a few protoscoleces were preserved in 70% ethanol for molecular analysis.

## Molecular analysis

Total genomic DNA was extracted from a single *Echinococcus* protoscolex according to the protocol described by Tkach and Pawlowski [7]. Since the mitochondrial nad1 gene has long been a target of choice for differentiation among *Echinococcus* genotypes and is well represented in the GenBank, we amplified a fragment at the 3’ end of the nad1 gene from our isolate by PCR using forward primer JB11 (5’− AGA TTC GTA AGG GGC CTA ATA − 3’) [8] and reverse primer Cyclo-trnNR (5’− TTC YTG AAG TTA ACA GCA TCA − 3’) [9]. PCR amplification was carried out in a total volume of 25 µl using One-Taq quick load PCR mix from New England Biolabs (Ipswich, Massachusetts, USA) according to the manufacturer’s instructions; annealing temperature was 45 °C. PCR product was visualized using agarose gel electrophoresis. Illustra ExoProStar PCR clean-up enzymatic kit from Cytiva (Marlborough, Massachusetts, USA) was used to purify PCR products and BrightDye Terminator Cycle Sequencing Kit (MCLAB, California, USA) was used to cycle-sequence purified PCR product. Sequencing reaction product was cleaned using BigDye Sequencing Clean Up Kit from MCLAB and subsequently run on an SeqStudio Genetic Analyzer (Thermo Fisher Scientific, Waltham, Massachusetts, USA). The PCR primers and additional internal primer JB12 (5’− ACC ACT AAC TAA TTC ACT TTC − 3’) were used for sequencing [8]. Contiguous sequence was assembled using Sequencher 4.2 software (GeneCodes Corp., Ann Arbor, Michigan, USA). Upon trimming of the primer regions, the newly generated sequence was deposited in GenBank under the accession number QB140010. NCBI BLAST search was used to compare our sequence with previously sequenced isolates of *Echinococcus* spp.

The obtained nad1 sequence was 765 nucleotides long. It proved to be almost identical (only 1 nucleotide difference) to multiple sequences of G1 genotype of *Echinococcus granulosus* s. str., mostly from sheep and cattle in Turkey, Iran and Armenia. Likewise, our sequence showed only 2 nucleotide differences from sequences of G1 genotype from distant geographic regions and different intermediate hosts, e.g., pigs in Argentina. This is the evidence of a highly homogenized genome of *E. granulosus* resulting from extensive movement of its definitive and intermediate hosts around the world over a long period of time.

## Discussion

The life cycle of *E. granulosus sensu stricto* is mainly anthropic, perpetuating by transmission between domestic dogs and livestock, particularly sheep. However, CE could be spilled over to wild mammals, mainly ungulates, from dog-livestock transmission system [10]. As far as we know, CE has not been reported in NHPs in the wild, likely because of the difficulty of sampling and examination of wild animals, especially NHP, in the nature [10]. In contrast, many publications have reported the disease in captive primates, including rhesus macaques, pig-tailed macaques, red-tailed guenons, ring-tailed lemurs, red-ruffed lemurs, baboons, and great apes [11–17].

This is the first report of *E. granulosus sensu stricto* genotype G1 in a common chimpanzee in Kuwait and the first case of *Echinococcus* in a chimpanzee accompanied by genotyping. Previously, in a zoo in California, a chimpanzee was reported to be infected with a hydatid cyst, perforating its abdominal wall; however, no other clinical or pathological data were provided, and the cyst was not genotyped [11].

Despite several previous reports of CE in captive NHPs, the genetic data characterizing *E*. *granulosus sensu lato*, in NHPs is very limited. In addition to the present case, genotype G1 has been reported in other primates, e.g., ring-tailed lemurs (*Lemur catta*) and a red-tailed guenon (*Cercopithecus ascanius*) [15, 16]. In UK, genotype G5 (*E.ortleppi*) was detected in one ring-tailed lemur and genotype G4 (*E. equinus*) was documented in both red ruffed lemur (*Varecia rubra*) and ring-tailed lemur [15, 17]. Based on reports from lemurs, these authors cautiously suggested that *E. equinus* may have zoonotic potential. However, Thompson and Smyth [18] failed to infect rhesus macaques experimentally by eggs obtained from adult cestodes with horse/dog cycle. Based on these data, there is difference in susceptibility to *E. equinus* between rhesus macaques and lemurs. Therefore, assumption that *E. equinus* may infect humans is premature.

The source and initial time of infection in case described herein could not be determined with certainty. We suggest three possibilities: (1) The animal may have been exposed to the infection via the feces of the final hosts (canids). However, results of screening of the feces of the wild canids in the zoo for the presence of taeniid eggs using floatation technique were negative. (2) The chimpanzee might have acquired the infection in Nigeria before its arrival in Kuwait 25 years ago. Little is known about the duration of infection and the time point of the appearance of clinical symptoms in NHPs [19]. Hydatid cyst could be detected in experimentally infected vervet monkeys and baboons 15–28 months post-infection. Assuming that the incubation period of hydatid cyst in chimpanzee is comparable to the time frame observed in humans, it could last many years [1]. However, our previous report of echinococcosis in a vervet monkey, which was born and raised in the same zoo [20], suggests a possibility of the chimpanzee infection upon arrival in Kuwait. (3) Probably, the source of infection could be via contaminated food brought from the local market. Federer et al. [21] found that vegetables and fruit contaminated with taeniid eggs from markets in Europe posed a threat to captive primates. The same can be true in Kuwait.

Stray dogs infected with *E. granulosus*-infected are the most likely source of the contamination of vegetables in Kuwait. Hassounah and Behbehani [6] found that 23% of examined stray dogs were infected with *E. granulosus*. Among livestock, sheep have been documented to be the most important intermediate hosts for this cestode in Kuwait [22]. Our finding of the genotype G1 (sheep genotype) in the chimpanzee supports this conclusion.

*Echinococcus granulosus sensu stricto* genotype G1 is common in Middle East countries, indicating comparable epidemiological situation in these countries where socioeconomic factors, human activities and sheep husbandry practices facilitate *E. granulosus* transmission. In Kuwait, the majority of sheep are free-range, moving freely to graze which allows them to be in extensive contact with shepherd dogs in the desert and remote areas around cities. Stray and guard dogs often access livestock farms and backyard gardens.

Mutton is the preferred meat in Kuwait and home slaughtering is common, particularly on religious and social occasions in the desert and remote areas, where Bedouins feed dogs with sheep entrails. In addition, the bodies of dead sheep are often left in the desert which make them available not only to dogs but also to wild canids. This scenario may result in sylvatic life cycle of *E. granulosus*; however, this point needs further investigation. It has been found that the high prevalence of *E. granulosus* infection in dogs [6] is correlated with the frequently reported human CE cases [23] in the Bedouin’s communities.

Extensive contact and habitat overlap between sheep and dogs increases the probability of contamination of the environment with eggs of *E. granulosus* and creates a higher risk of infection for accidental hosts, e.g., primates. The report of CE in a zoo chimpanzee further emphasizes the issue of environment contamination. Shweiki et al. [5] estimated the incidence of CE in humans in Kuwait at the rate of 3.6 cases per 100,000; they considered this figure to be an underestimation of the true incidence.

The lesions and symptoms caused by the growing hydatid cyst are determined by its size, site and condition [17]. In the present case, the large size of the cyst and its localization in the umbilical fissure of the liver, were determinants for inducing lesions and clinical signs. This is a typical case of hepatic cystic echinococcosis, comparable with that observed in humans [24].

As *E. granulosus* infection usually remains asymptomatic for months or years and the symptoms appear at the latest stages of the disease, the antemortem diagnosis is difficult and the prognosis is bad. Therefore, it is recommended that the apparently healthy captive primates in the zoo are examined serologically and if necessary, by radiology to diagnose CE cases early enough for suitable medical intervention.

## Conclusion

We report the first case of *E. granulosus sensu stricto* genotype G1 in a common chimpanzee in the Kuwait Zoo. This is the first study to characterize hydatid cyst from humans or animals in the country using DNA sequencing and also the first genetically confirmed case of the genotype G1 in a chimpanzee. The infection of a captive chimpanzee with CE highlights the issue of environment contamination and highlights the need for regular monitoring to have up to date information regarding the prevalence of CE and *Echinococcus* in livestock, as well as domestic and wild canids in Kuwait.

## Data Availability

Newly obtained sequence has been deposited in GenBank and is publicly accessible.
